# Reading fluency and statistical learning across modalities and domains: Online and offline measures

**DOI:** 10.1371/journal.pone.0281788

**Published:** 2023-03-23

**Authors:** Ágnes Lukács, Dorottya Dobó, Ágnes Szőllősi, Kornél Németh, Krisztina Sára Lukics

**Affiliations:** 1 Department of Cognitive Science, Budapest University of Technology and Economics, Budapest, Hungary; 2 MTA-BME Momentum Language Acquisition Research Group, Eötvös Loránd Research Network (ELKH), Budapest, Hungary; 3 Institute of Cognitive Neuroscience and Psychology, Eötvös Loránd Research Network (ELKH), Budapest, Hungary; 4 Centre for Cognitive Medicine, University of Szeged, Szeged, Hungary; University of Padova, ITALY

## Abstract

The vulnerability of statistical learning has been demonstrated in reading difficulties in both the visual and acoustic modalities. We examined segmentation abilities of Hungarian speaking adolescents with different levels of reading fluency in the acoustic verbal and visual nonverbal domains. We applied online target detection tasks, where the extent of learning is reflected in differences between reaction times to predictable versus unpredictable targets. Explicit judgments of well-formedness were also elicited in an offline two-alternative forced choice (2AFC) task. Learning was evident in both the acoustic verbal and visual nonverbal tasks, both in online and offline measures, but learning effects were larger both in online and offline tasks in the verbal acoustic condition. We haven’t found evidence for a significant relationship between statistical learning and reading fluency in adolescents in either modality. Together with earlier findings, these results suggest that the relationship between reading and statistical learning is dependent on the domain, modality and nature of the statistical learning task, on the reading task, on the age of participants, and on the specific language. The online target detection task is a promising tool which can be adapted to a wider set of tasks to further explore the contribution of statistical learning to reading acquisition in participants from different populations.

## 1. Introduction

As learning through reading is central in most school settings, difficulty in reading acquisition is causal in problems with learning and affects academic progress of students. It has recently been suggested that reading problems might result from deficits in some type of basic learning mechanism [1–3], more specifically from deficits in statistical learning [4]. Statistical learning is the capacity to extract patterns and regularities from environmental stimuli through observation sensitive to frequency distributions and transitional probabilities [5]. This learning mechanism is argued to be essential in many areas of cognition across domains and modalities, including discrimination, categorization, segmentation, and prediction [5, 6]. Given the nature and complexity of human languages, statistical learning is suggested to play a key role in the acquisition of many levels of language [5, 7, 8]: in developing phoneme representations (tuning phoneme representations to the mother tongue), in word segmentation, pairing words and meanings, and in extracting grammatical regularities. Indeed, associations between statistical learning and performance on different language tasks have been found including a) speech perception in noise [9, 10], b) adult natural language ability [11], c) natural language processing in infants [12], d) syntactic priming effects in preschool children [13], e) syntax comprehension [14, 15], and f) lexical access [16]. Statistical learning and language processing also show a neural overlap [17–19]. Altogether, the above results suggest that statistical learning shares mechanisms with language processing and language learning on many levels.

The contribution of statistical learning is not restricted to spoken language acquisition and use. It may have an impact on learning to read both in direct and indirect ways: 1) in the extraction of orthographic regularities, 2) in the acquisition of orthography-phonology correspondences, 3) and it can also influence the efficiency of the reading process indirectly through contributing to reading-related skills like word learning and grammar acquisition [20–26]. Based on these observations, it is expected that impairments in statistical learning abilities are associated with reading difficulties.

The most prominent form of reading difficulty is developmental dyslexia, a neurodevelopmental disorder involving the selective impairment of reading, which is not caused by reduced intelligence, low socio-economic status or inadequate teaching methods [27]. Prevalence estimates vary between 5–17% in the US [28, 29], but they can greatly vary across countries and languages depending on diagnostic criteria and orthography type (see e.g., [30, 31]). At the behavioural level, developmental dyslexia is defined by reading difficulties, explained by a combination of well-documented deficits in phonological processing difficulties, and lexical retrieval problems and reduced capacity of verbal short term memory [32–40].

In line with the expectations outlined above, statistical learning has been shown to be vulnerable in dyslexia and recent studies have found evidence for the association of statistical learning deficit and reading impairment in this developmental disorder [28, 41, 42]. Impairments have been observed in different forms of statistical learning in visuomotor, visual, and acoustic tasks. Motor sequence learning [43–46], word segmentation [28, 42, 47] and artificial grammar learning tasks [41, 48–51] are all vulnerable to statistical learning deficits in dyslexia.

However, results are far from unequivocal and methods vary greatly. For example, van Witteloostuijn, Boersma, Wijnen, and Rispens [52] have not found a deficit in statistical learning either in the acoustic or the visual modality in a large sample of Dutch speaking children with dyslexia using three different tasks. As a further example, while meta-analyses show evidence for a deficit in motor sequence learning in the Serial Reaction Time (SRT) task [53], evidence for a visual statistical learning deficit is less straightforward: a meta-analysis of visual artificial grammar learning studies in dyslexia [54] found evidence for deficits in visual artificial grammar learning, but the authors point out that if publication bias against negative results is taken into account, the effect could be zero (see also [4], on other methodological problems). These meta-analyses also show that age is a further significant factor: statistical learning impairment is more prominent in children than in adults on both visuomotor and visual tasks.

Since phonological processing, segmentation, and awareness are acoustic skills that are critical for successful reading acquisition, testing statistical learning in the acoustic modality is also important in exploring mechanisms of reading acquisition and reading impairment. However, so far acoustic statistical learning has received much less attention in dyslexia than the learning in the visual modality. Dobó and colleagues [48] found less efficient artificial grammar learning in dyslexia with verbal acoustic stimuli. Kahta and Schiff [41] tested artificial grammar learning in the acoustic modality with musical tones in adults with dyslexia, and observed deficient learning. Statistical segmentation abilities also show deficits in different domains and modalities. Relying on the word segmentation paradigm [55], Gabay and colleagues [28] found deficient learning in high-functioning university students with dyslexia relative to age and IQ matched controls with both linguistic and non-linguistic stimuli in the acoustic modality with very brief (< 3-minute) training and an explicit 2AFC test, although both groups displayed above chance learning levels. They also observed significant correlations between learning indices in the two modalities (verbal and visual) and several measures of reading ability.

The above review, as the majority of studies examining the relationship of statistical learning and reading, focused on dyslexia, but statistical learning seems to support reading acquisition in all readers. Reading skills show considerable variability in the typical population as well [56–59], influenced by several cognitive and socioeconomic factors [50, 60–62]. Statistical learning is one of the contributors: correlation studies have demonstrated important associations between statistical learning, language processing and reading abilities (with accuracy, fluency and decoding,) [21] in both children and adults [26] in typical readers.

To sum up, statistical learning plays an important role in spoken and written language acquisition and use. Impairments of this learning mechanism seem to be present in the case of reading difficulties, for instance, in developmental dyslexia. However, the small number of findings so far have not supported the vulnerability of statistical learning in reading impairments unequivocally. Also, there are very few studies that investigate the full variety of reading abilities along a spectrum.

In the present study, we aimed to investigate the complex relationship between statistical learning, more specifically statistical segmentation, and reading skills. While most previous studies examined segmentation in the acoustic modality in relation with reading difficulties, visual segmentation skills, which are also relevant for reading, received less attention. Therefore, our aim was to test and directly compare statistical learning in verbal and visual segmentation tasks.

Based on the empirical findings related to reading difficulties and dyslexia, deficits in statistical learning can occur in both the visual and the acoustic modalities. As statistical learning operates on modality-specific representations with modality-specific constraints [5, 6], impairment of this learning mechanism in different modalities can contribute to the reading difficulties independently. Segmentation abilities are important in reading development in both the acoustic and visual modalities. In the acoustic modality, deficits in phonological awareness involving the ability to segment linguistic stimuli into smaller components (most often words into syllables and phonemes) are frequently associated with reading difficulties [34, 37, 41, 63–65]. Segmentation in the visual modality is less obviously relevant, as word boundaries in reading are clearly marked by spaces. However, skilled readers rely on recognizing frequent and reliable sublexical chunks of graphemes [24]. Visual segmentation and fast recognition of prefixes also benefit the reading of morphologically complex words, especially in morphologically rich languages like Hungarian. Besides, the association of statistical learning and reading ability in different modalities has been mostly confirmed in studies of speakers of languages with opaque orthographies (such as Chinese, Hebrew and English), and less is known about this association in languages with transparent orthographies (such as Hungarian). As Hungarian has shallow orthography, readers might rely on the phonological route more than the lexical route in reading compared to languages with more opaque orthographies [66–68], making acoustic and visual pattern learning abilities similarly important in reading. These factors make testing segmentation abilities both in the acoustic and visual modalities well motivated.

Beyond controversial findings based on diverse methods, our study was also motivated by methodological concerns about the efficiency of explicit offline measures of statistical learning from judgement tasks [69–71]. In most experimental designs, after the training phase participants are required to explicitly reflect upon the grammatical well-formedness of artificial strings in some form. Such tests yield explicit measures building on metalinguistic awareness, which can distort results and may not truly reflect the effectiveness of the learning process in statistical learning, especially in neurodevelopmental disorders, where memory and metacognitive abilities required for the judgement task may themselves be impaired. In online versions of statistical learning tasks, participants become more engaged in learning, e.g. by having to press a key when they hear a syllable (syllable monitoring). During the training phase, target patterns become implicitly more and more predictable resulting in quicker button presses. The time between the appearance of the stimulus and the response is informative of the learning process: as participants learn the pattern, reaction times shorten.

Motivated by the necessity of measures that are more sensitive to learning and individual differences, and to gain a more accurate picture of the statistical learning in different domains in reading impairments, we aimed to test both visual and acoustic statistical learning relying on both online and offline measures (following Lukics and Lukács [70]). Since reading difficulties are not exclusive to individuals with dyslexia, and due to the lack of clear-cut boundaries and comprehensive standardised diagnostic tools for dyslexia in adolescents in Hungarian, we recruited adolescents with dyslexia for our study, but we did not compare the performance between participants with dyslexia and without dyslexia. Instead, as a methodological novelty, to gain better insight into the relationship of statistical learning and reading skills, we treated reading skills as a continuum with impaired and good reading fluency at the two ends of the spectrum. To explore their relationship with reading fluency, we measured acoustic verbal and visual nonverbal segmentation abilities in online target detection tasks, where the extent of learning is reflected in the differences between reaction times to predictable versus unpredictable targets. At the same time, explicit judgments of well-formedness were also elicited in an offline 2AFC task. As verbal short term and working memory are important determinants of reading proficiency, and they may also influence performance in the verbal statistical learning task, we assessed them to control for their effects on the relationship of statistical learning and reading performance. To test similar relationships with nonverbal visual statistical learning, we also assessed visual working memory. We formulated the following hypotheses:

1. Statistical learning will be evident and equally efficient in the acoustic verbal and the visual nonverbal conditions.
2. Both online and offline measures will demonstrate significant learning effects.
3. Both acoustic and visual statistical learning will be positively associated with reading fluency, and there will be no difference in the strength of relationship between domains.
4. Both online and offline measures will show significant relationships with reading fluency. Since they are less confounded by other factors, and thus are more sensitive to statistical learning than 2AFC scores, online measures will have stronger associations with reading fluency.

## 2. Materials and methods

Materials for the experiment, raw and processed data, and scripts for data analysis are available here: https://osf.io/xmzd8/?view_only=f8c544ca3ce541b9ab901044ed991b29.

### 2.1. Participants

83 Hungarian adolescents (10 females, mean age = 17.7, SD = 1.2) participated in the study. They were all native speakers of Hungarian. Participants were tested with the informed consent of their parents’ together with their own informed consent, except for participants above 18 years of age who were tested only with their own informed consent in accordance with the principles set out in the Declaration of Helsinki and the stipulations of the local Institutional Review Board. The study was approved by the United Ethical Review Committee for Research in Psychology (approval number: EPKEB-2018/87). Participants and parents gave their written consent to participation and data collection.

Participants were recruited from three secondary schools in Budapest. One of these institutions was a school in which students with learning difficulties were integrated, while the other two were mainstream secondary schools. We recruited participants with reading difficulties from the integrating school to cover all the spectrum of reading abilities. Students were chosen based on the expert opinion of a speech-therapist from the institution, some of them having a previous diagnosis of developmental dyslexia. Since reading fluency is the most reliable indicator of reading disorders in both opaque [72–74] and transparent languages [75], we applied a reading fluency task to measure reading skills uniformly both in participants with a previous diagnosis of developmental dyslexia and in participants without a diagnosis and/or reading problems.

**Fig 1:**
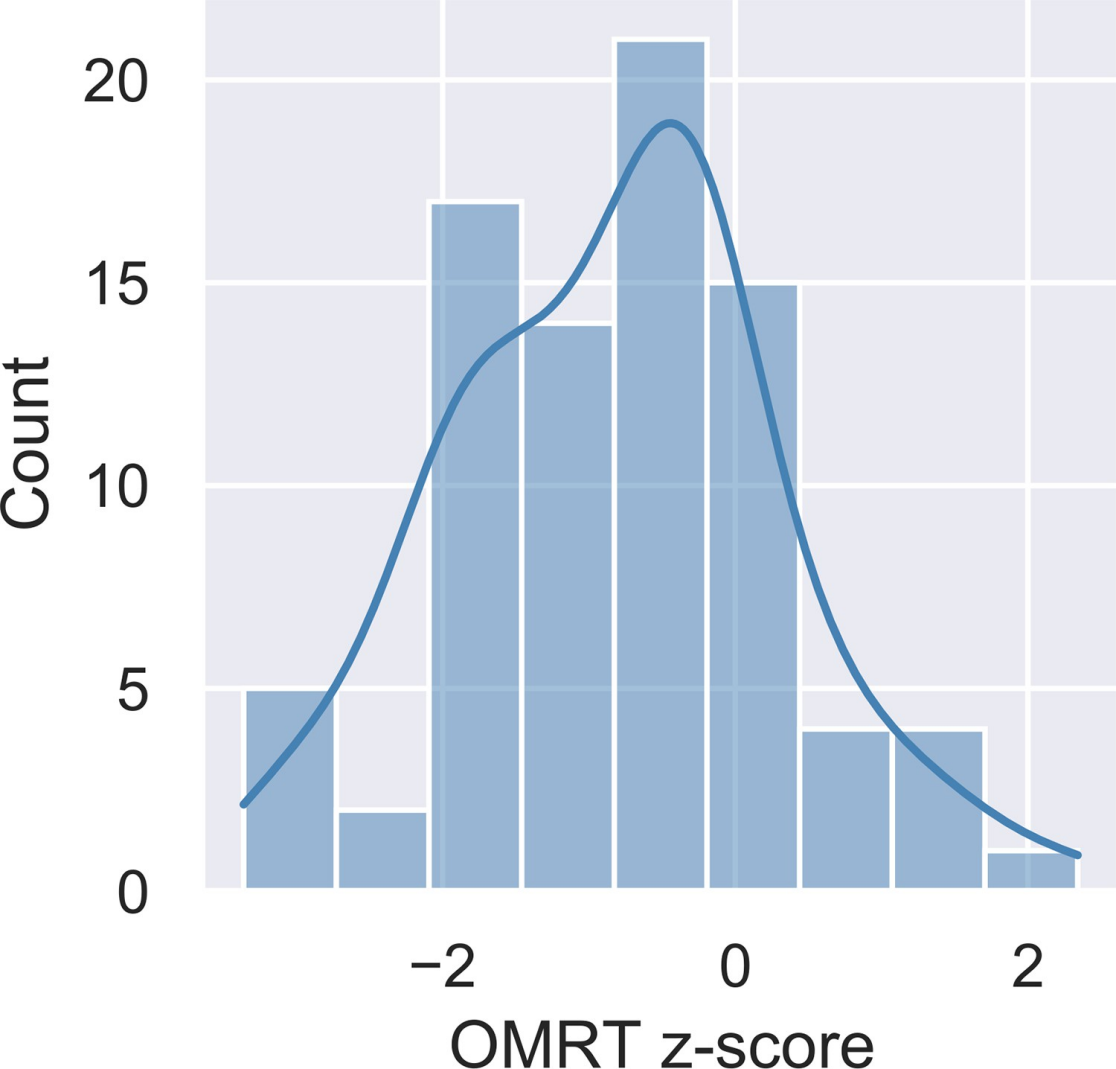
Distribution of the *OMRT z-score* in the sample.

In the reading fluency task, participants were given a printed text on paper and were asked to read it aloud. The reading index was the number of syllables read in 60 seconds (One Minute Reading Task, OMRT [76]). Raw syllable counts were normalized based on data from a larger sample of adolescents. Fig 1 illustrates the distribution of normalized OMRT scores in the sample. We also included other background measures investigating short term and working memory, which might have potentially influenced results of statistical learning tasks, or modulate the relationship between reading ability and statistical learning (forward and backward digit span, non-word repetition task; Hungarian versions: Racsmány, Lukács, Németh, and Pléh [77], nback tasks with letters and fractal images).

Data was missing in the case of seven participants for the online segmentation tasks, in the case of three participants for the offline segmentation tasks, and in the case of one participant for the digit span tasks. To maximize statistical power, we decided to include participants whose data were missing in some tasks, and performed pairwise exclusions in statistical analyses where possible.

### 2.2. Stimuli

The segmentation tasks were modelled after Saffran and colleagues’ seminal studies [55, 78]. Besides the traditional tests, the current version of the paradigm includes an online target detection task to detect the acquisition of statistical patterns during the learning phase [70] (see also [71, 79, 80] for similar designs). In addition to the acoustic verbal version of the task, we also administered a visual nonverbal version with the same design.

#### 2.2.1. Verbal acoustic segmentation task

Stimuli in the verbal acoustic segmentation task consisted of 12 recorded nonsense consonant-vowel syllables (cé /ʦeː/, de /dɛ/, gá /gaː/, go /go/, ha /hɒ/, ki /ki/, mü /my/, pe /pɛ/, sá /ʃaː/, tu /tu/, ta /tɒ/, vi /vi/) spoken by a male Hungarian speaker. Syllables were recorded in isolation to prevent coarticulation effects, and were manipulated in Praat. As a result, all recordings had a pitch of 130 Hz, a length of 400 ms, and maximum intensities between 76.6 and 83.5 dB. Sound intensities of syllables were judged to be equal by two independent listeners, resulting in monotonous stimuli which did not provide any prosodic cues to segmentation. For the online training phase, syllables were structured into five streams. In four of these streams, syllables were organized into four artificial words (*cégávi*, *detamü*, *hagoki*, *sápetu*), which followed Hungarian phonotactics but are not existing words in Hungarian. These words followed each other in a pseudorandom order with the constraint that the same word could not occur twice subsequently (word streams). In these word streams, transitional probabilities (TPs) within words were 1, while TPs at word boundaries were 0.33. In a fifth stream, individual syllables followed a pseudorandom order in a way that two syllables could not occur twice subsequently (random stream). In all of the streams, syllables were presented with pauses of 120 ms between them, resulting in a presentation rate of 520 ms (the duration of pauses, thus the presentation rate, was a feature of the E-Prime software which we could not modify). The only cues for word boundaries were TPs. In each stream, 180 syllables were presented, each syllable with an equal frequency of occurring 15 times within each block, both in word and random streams.

For the 2AFC task, we used the four words from the training streams. In addition, we created four part-word (syllable triplets spanning a word boundary in the word streams), and four non-word (syllable triplets not occurring together in the word streams) foils. Stimuli for the verbal acoustic task are provided in Table 1.

**Table 1:** Words, part-words and non-words used in the 2AFC task.

Words	Part-words	Non-words
cégávi	kicégá	céhatu
detamü	mühago	dekisá
hagoki	tudeta	gápego
sápetu	visápe	mütavi

#### 2.2.2. Nonverbal visual segmentation task

Stimuli for the nonverbal visual task consisted of 12 monochrome (black and white) symbols that were difficult to verbalize. The vertical size of stimuli was 3 cm, and participants viewed them from a distance of approximately 60 cm from the screen. The 12 symbols were organized into four words, and these words followed each other in a pseudorandom order with the constraint that a given word could not occur twice in a row (word streams). Altogether, participants were presented with four word streams, where TPs within words were 1, while TPs on word boundaries were 0.33. In a fifth stream, individual symbols followed each other in a pseudorandom order with the constraint that a given symbol could not occur twice in a row (random stream). In each stream, 180 symbols were presented, each symbol with an equal frequency of occurring 15 times within each block, both in word streams and in the random stream. Symbols were presented at a presentation rate of 420 ms (the duration of pauses between symbols, 20 ms, was a feature of the E-Prime software which we could not modify, so we added it to the presentation rate).

**Fig 2:**
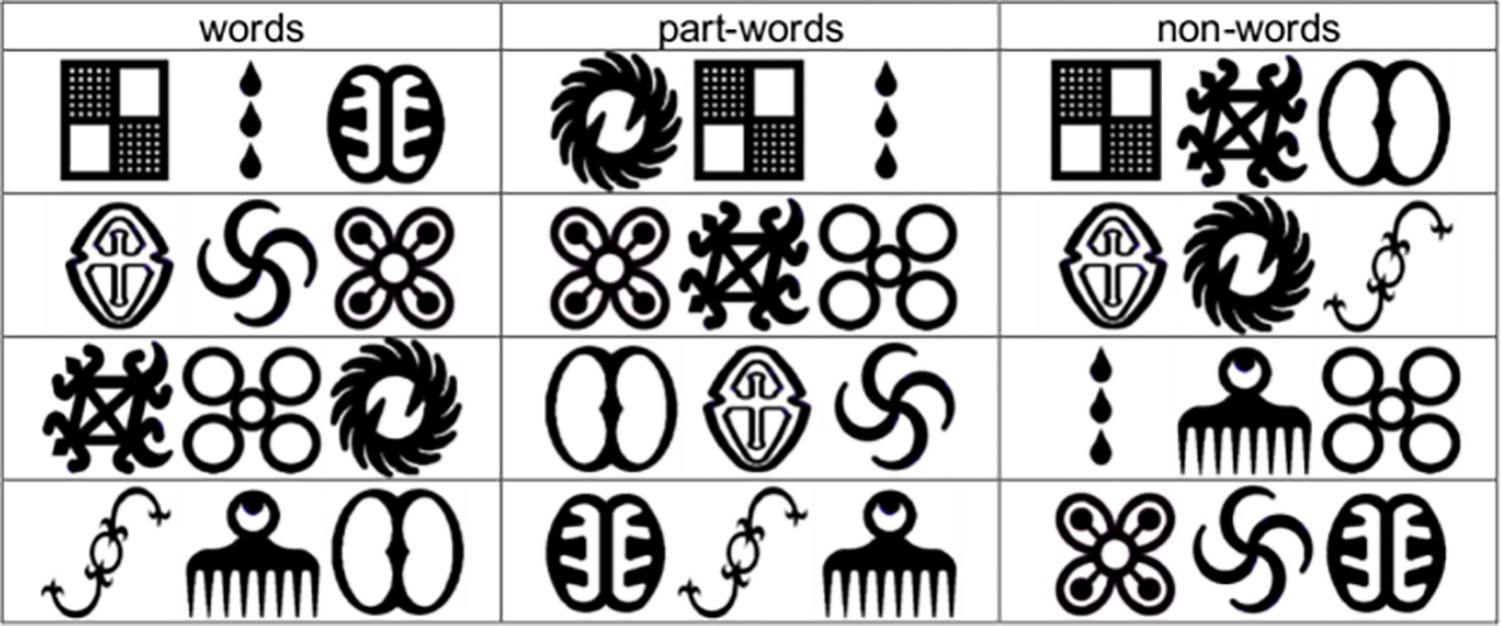
Stimuli in the nonverbal visual task.

For the 2AFC task, we used the four words from the training, and we created four part-word (symbol triplets spanning a word boundary in the word streams), and four non-word (symbol triplets not occurring together in the word streams) foils. Stimuli for the nonverbal visual task are provided in Fig 2.

### 2.3. Task and procedure

Tasks were administered in silent classrooms of the schools in small groups. Acoustic stimuli were presented over headphones and the tests were recorded using the E-Prime 2.0 Professional software (Psychology Software Tools Inc., Pittsburgh, PA, USA).

Participants listened to stimuli through headphones in the verbal acoustic task, and viewed stimuli on the computer screen in the nonverbal visual task. The first phase of both tasks was an online learning phase based on the design of the Serial Reaction Time Task [81], aimed at tapping into the online process of learning. During listening to the speech stream or watching the symbol stream, participants’ task was to respond to a target syllable or symbol (the same item throughout the entire online session). This target was the last item of one of the four three-item words, counterbalanced across participants. Last items were chosen as targets because previous work has shown that in target detection tasks participants show the most reliable learning effect in RT decreases to the last syllables of words in speech segmentation tasks as opposed to middle-position syllables [80, 82]. The online training phase consisted of three streams in three training blocks (Blocks TRN1 to TRN3). Each training stream consisted of 60 words, so altogether, 180 words were presented during training; each of the four words were presented 45 times. In the training blocks we measured how efficiently participants predict the target syllable or symbol through the course of learning, reflected in the changes in reaction times and accuracies over time. We also included two test blocks in the online tasks. The first test block was the random stream, where the word structure was disrupted, and syllables or symbols followed each other in a pseudorandom order (Block RND4). Block RND4 was the baseline or reference block, as the target item was not predictable, so RTs and accuracies were only influenced by the general practice effect in the task. The second test block was a word stream again, serving as a recovery block (Block REC5). Throughout the five blocks, participants performed an item-detection task: they were instructed to respond by pressing the spacebar every time they heard or saw the target (specified in the instruction) as quickly and accurately as they could, and to press the key ‘A’ in the case of any other syllable or symbol.

After the online task, offline learning measures were collected from a 2AFC task. In both acoustic verbal and visual nonverbal tasks, participants were presented with 24 pairs of three-item sequences. We tested three types of contrasts here: word vs. part-word, word vs. non-word, and part-word vs. non-word sequences. Including different contrast types allows testing statistical learning with different levels of sensitivity: the word vs. part-word contrast shows how sensitive participants are to differences between stronger compared to weaker TP distributions in triplets (TP1 = 1; TP2 = 1 vs. TP1 = 0.33; TP2 = 1), the word vs. non-word contrast shows how sensitive participants are to differences between strong TP sequences over sequences with zero TPs (TP1 = 1; TP2 = 1 vs. TP1 = 0; TP2 = 0), and finally, the part-word vs. non-word contrast is informative about whether participants favor even weaker TP sequences over sequences with zero TPs (TP1 = 0.33; TP2 = 1 vs. TP1 = 0; TP2 = 0). Moreover, by testing the ability to differentiate between items in different contrast types we wanted to increase the validity of our 2AFC measure. Sequences in each contrast were randomly selected for each participant, and the order of trigram types varied within contrasts. In each trial, participants heard or saw the two sequences, and they were instructed to indicate which one was more familiar based on the training streams. We quantified learning in accuracy rates: the number of correct answers divided by the number of all trials, yielding a number between 0 and 1.

## 3. Results

### 3.1. Segmentation tasks

In the online target detection tasks, RTs were collected for accurate button presses for targets within a -600 to 1200 ms time window from stimulus onset. Responses with negative time windows reflect anticipatory responses. As there is no uncertainty within words regarding the following syllable (TPs = 1), after learning, target syllables can be predicted with 100% certainty, so anticipatory responses are plausible (see e.g. Lammertink, Boersma, Wijnen, Rispens [83], who also included negative reaction times in their analyses). For each participant, we filtered responses with RTs outside the 1.5 interquartile range beyond the first and third quartiles of their RTs using Tukey’s fences criterion. To analyse reaction times, we calculated the median of RTs for each block by participant. We also report block accuracies.

**Fig 3:**
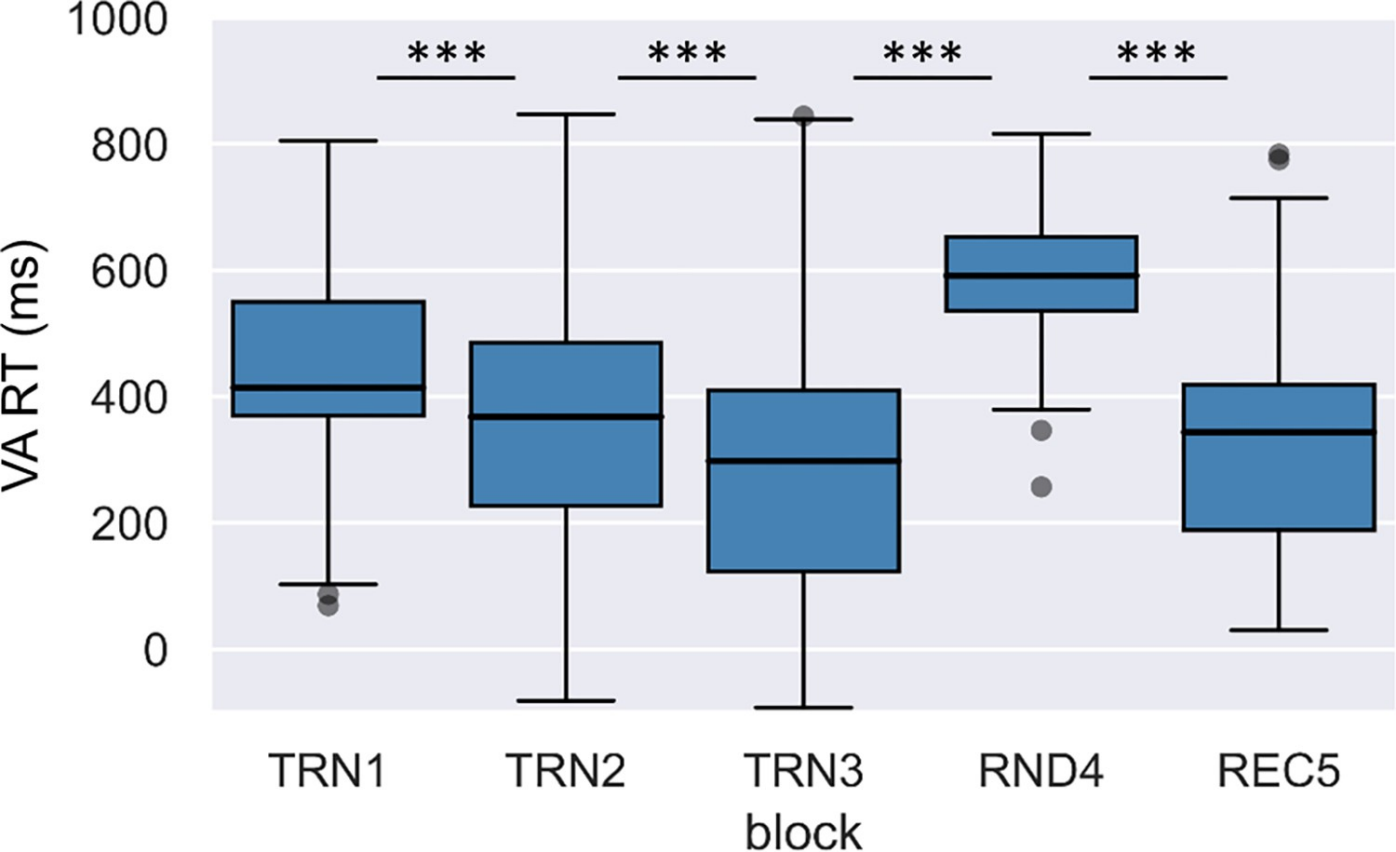
Change of median reaction times through blocks in the verbal acoustic condition. Boxes indicate reaction times between the first and third quartiles, horizontal lines indicate medians, whiskers illustrate the range of data, and individual dots outside the range represent outliers.

**Fig 4:**
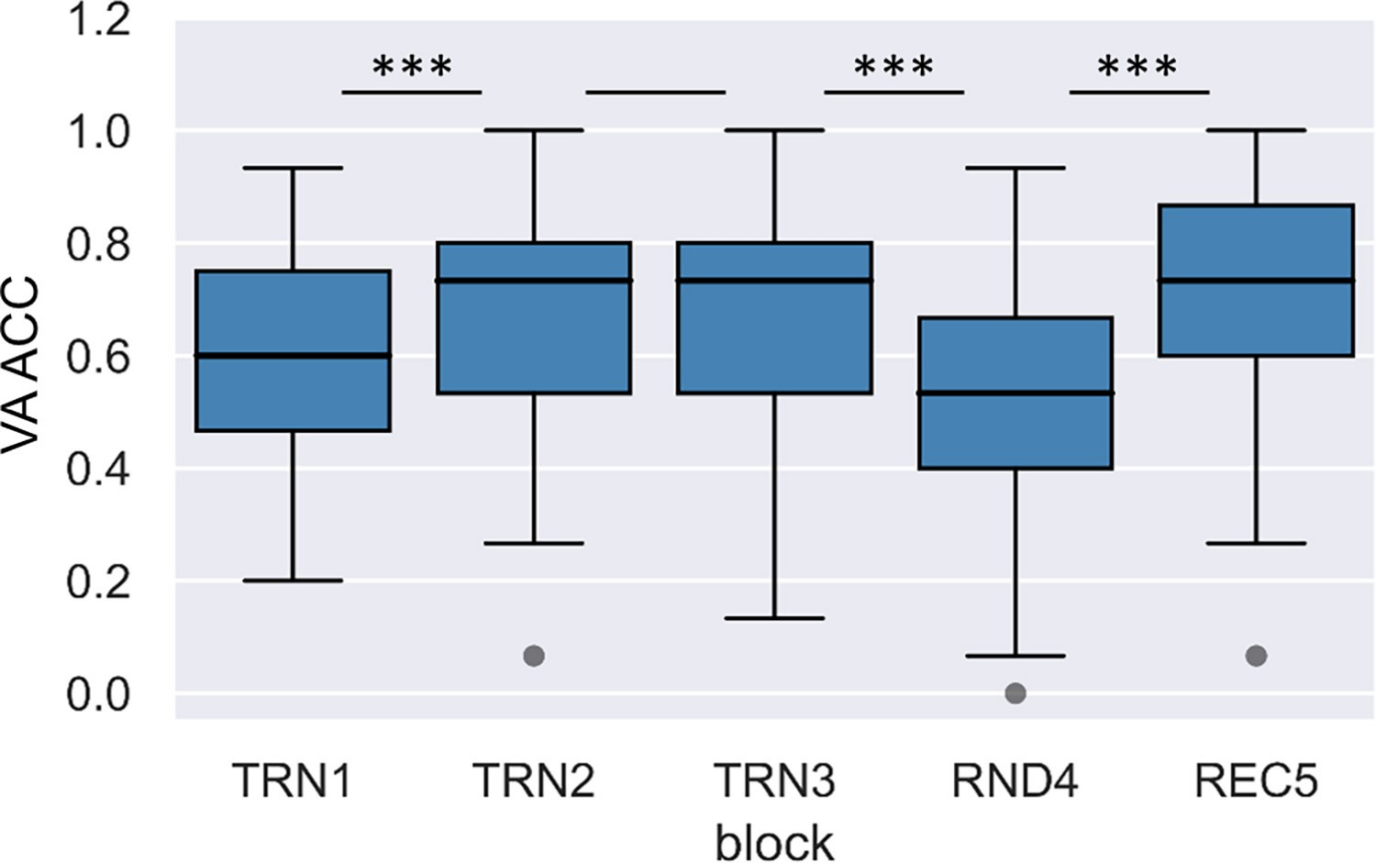
Accuracy rates across blocks in the verbal acoustic condition. Boxes indicate accuracy data between the first and third quartiles, horizontal lines indicate medians, and whiskers illustrate the range of data, and individual dots outside the range represent outliers.

Medians and ranges of RTs and accuracies by block in the verbal acoustic task are shown in Figs 3 and 4, respectively.

**Fig 5:**
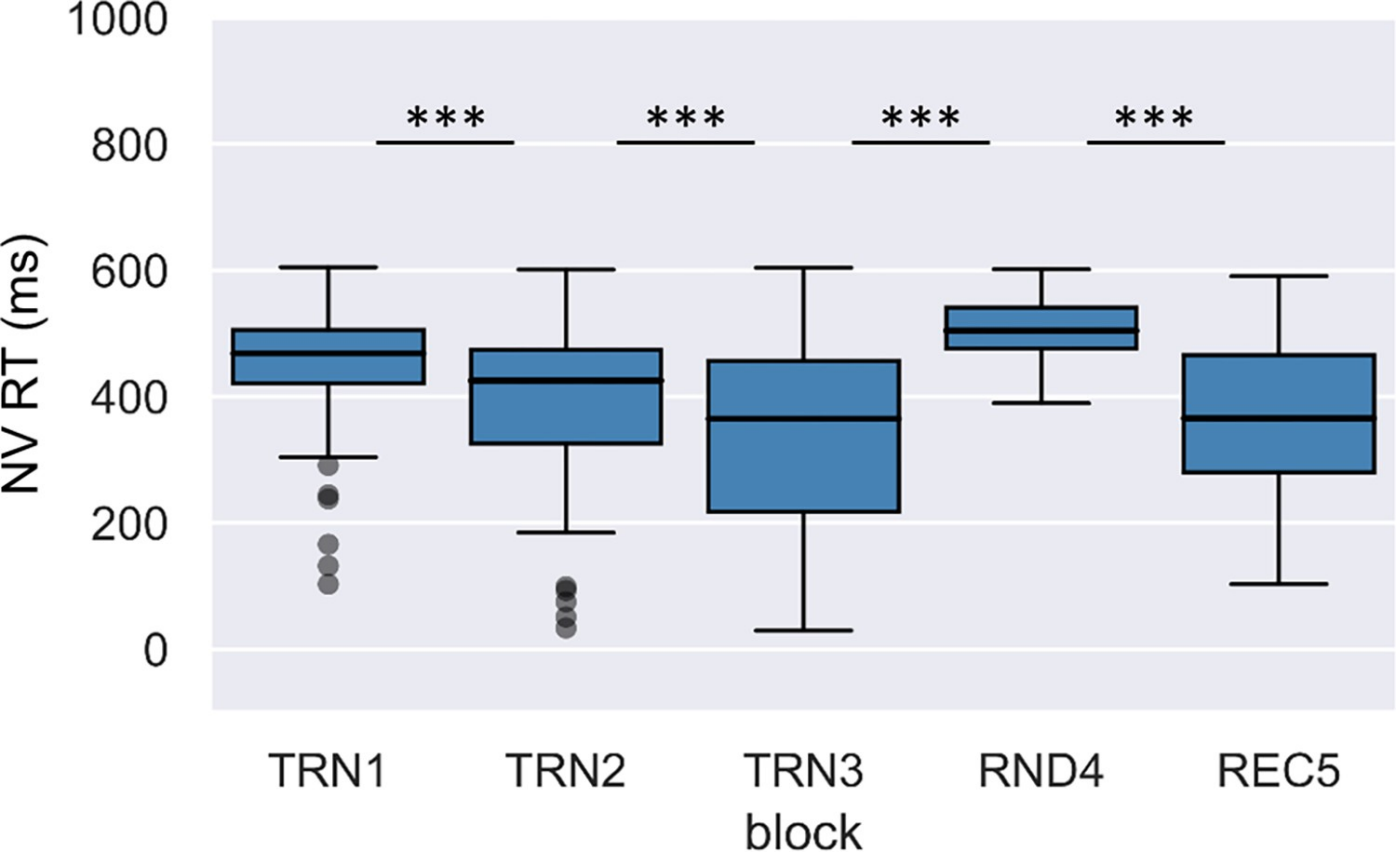
Change of median reaction times across blocks in the nonverbal visual condition. Boxes indicate RT data between the first and third quartiles, horizontal lines indicate medians, and whiskers illustrate the range of data, and individual dots outside the range represent outliers.

**Fig 6:**
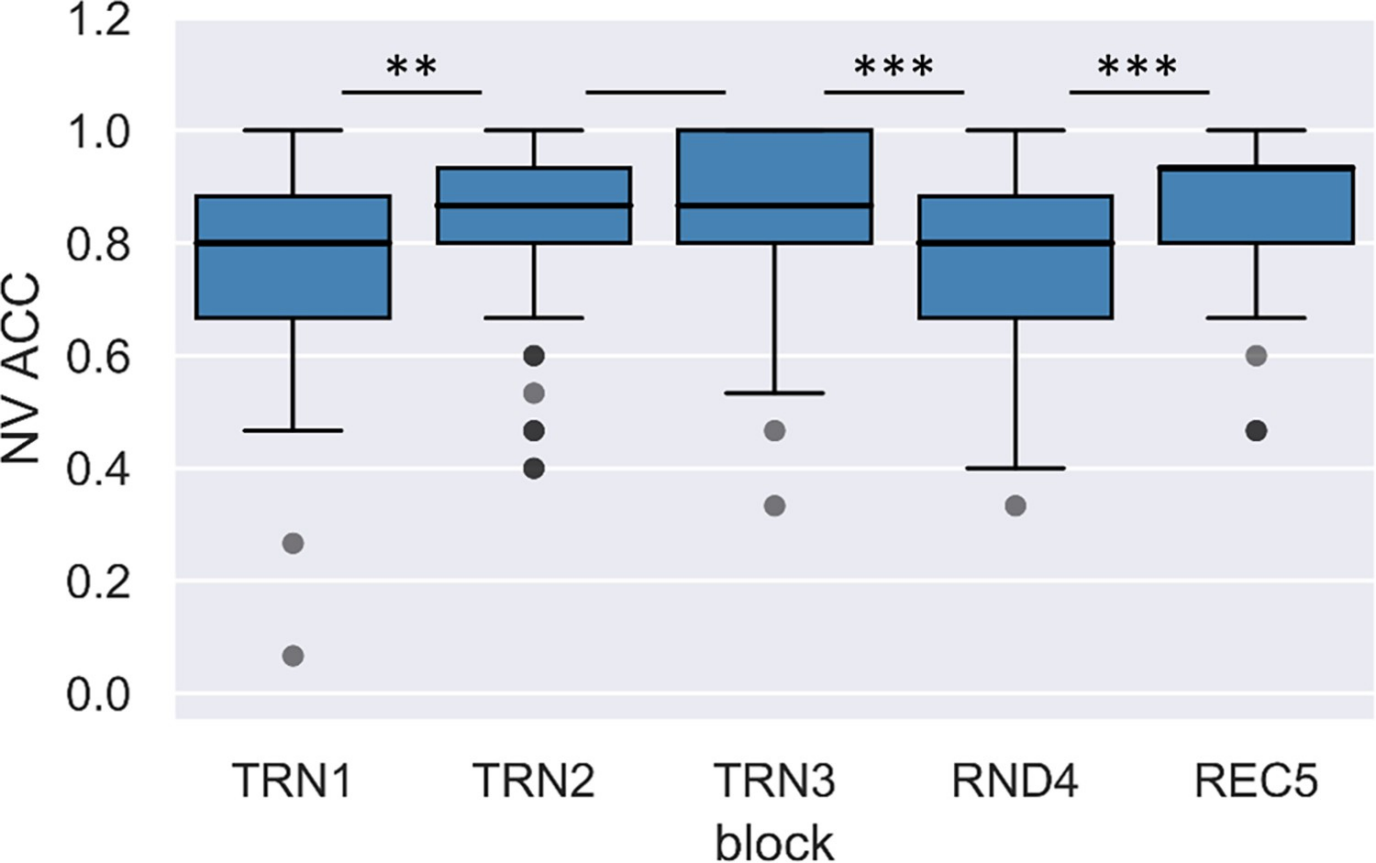
Accuracy rates across blocks in the nonverbal visual condition. Boxes indicate accuracy data between the first and third quartiles, horizontal lines indicate medians, and whiskers illustrate the range of data, and individual dots outside the range represent outliers.

Medians and ranges of RTs and accuracies by block in the nonverbal visual task are shown in Figs 5 and 6, respectively.

To investigate online learning through the segmentation tasks, we performed a two-factor repeated measures ANOVA with a Greenhouse-Geisser correction on RTs with Condition (verbal acoustic vs. nonverbal visual) and Block as within-subject factors. The effect of Condition was not significant, F(1,198.55) < 0.01, p = .965, ηp2 < .01. On the other hand, the effect of Block, F(2.84,198.55) = 124.54, p < .001, ηp2 = .64; and the interaction of Condition and Block were significant, F(2.80,198.55) = 7.23, p < .001, ηp2 = .09. Repeated contrast analyses revealed that neighboring blocks significantly differed from each other (Block TRN1 > Block TRN2: F(1,71) = 55.92, p < .001, ηp2 = .44; Block TRN2 > Block TRN3: F(1,71) = 43.53, p < .001, ηp2 = .38; Block TRN3 < Block RND4: F(1,71) = 253.41, p < .001, ηp2 = .78; Block RND4 > Block REC5: F(1,71) = 225.02, p < .001, ηp2 = .76). The tests of within-subject contrast for the Condition and Block interaction were only significant in the case of the contrast between the random and the neighboring word blocks (Block TRN1 > Block TRN2: F(1,71) = 1.45, p = .232, ηp2 = .02; Block TRN2 > Block TRN3: F(1,71) = 0.29, p = .593, ηp2 < .01; Block TRN3 < Block RND4: F(1,71) = 12.75, p = .001, ηp2 = .15; Block RND4 > Block REC5: F(1,71) = 12.56, p = .001, ηp2 = .15). To follow up on these interactions, we tested the effect of Block separately for the acoustic verbal and visual nonverbal conditions, both for TRN3 vs. RND4, and for RND4 vs. TRN5. The difference between reaction times between blocks TRN3 and RND4 was significant in both the acoustic verbal, t(74) = 12.04, p < .001, r = .81; and in the visual nonverbal condition, t(75) = 10.08, p < .001, r = .76. The difference between reaction times between blocks RND4 and REC5 were also significant in both the acoustic verbal, t(74) = 10.45, p < .001, r = .77; and in the visual nonverbal condition, t(75) = 10.38, p < .001, r = .77. Since all these comparisons showed significant differences, as a more direct way of comparing modality differences, we also calculated difference scores between blocks TRN3 and RND4, and blocks RND4 and REC5 in both conditions. We found that the reaction time differences between TRN3 and RND4, and RND4 and REC5 blocks were larger in the acoustic verbal than in the visual nonverbal condition (t(71) = 3.57, p = .001, r = .39; and t(71) = 3.54, p = .001, r = .39, respectively).

We also performed a two-factor repeated measures ANOVA with a Greenhouse-Geisser correction on accuracies with Condition and Block as within-subject factors. The effect of Condition, F(1,255.66) = 105.39, p < .001, ηp2 = .59; and the effect of Block were significant, F(3.37,255.66) = 25.75, p < .001, ηp2 = .26. The interaction of Condition and Block was also significant, F(3.55,255.66) = 2.60, p = .043, ηp2 = .04. Repeated contrast analyses revealed that there was only a significant difference between the first two blocks, and between the random block and the neighboring word blocks (Block TRN1 > Block TRN2: F(1,72) = 20.32, p < .001, ηp2 = .22; Block TRN2 > Block TRN3: F(1,72) = 1.76, p = .189, ηp2 = .02; Block TRN3 < Block RND4: F(1,72) = 42.49, p < .001, ηp2 = .37; Block RND4 > Block REC5: F(1,72) = 56.09, p < .001, ηp2 = .44). The tests of within-subject contrast for the Condition and Block interaction were only significant in the case of the contrast between the random and the recovery block (Block TRN1 > Block TRN2: F(1,72) = 0.39, p = .536, ηp2 = .01; Block TRN2 > Block TRN3: F(1,72) = 0.87, p = .355, ηp2 = .01; Block TRN3 < Block RND4: F(1,72) = 3.63, p = .061, ηp2 = .05; Block RND4 > Block REC5: F(1,72) = 5.05, p = .028, ηp2 = .07). To follow up on these interactions, we tested the effect of Block separately for the acoustic verbal and visual nonverbal conditions for blocks RND4 vs. REC5. We found that the reaction time differences between RND4 and REC5 were significant both in the acoustic verbal, t(75) = 5.97, p < .001, r = .57; and in the visual nonverbal conditions, t(75) = 4.94, p < .001, r = .50. Since these comparisons showed significant differences, as a more direct way of comparing modality differences, we also calculated accuracy difference scores between blocks RND4 and REC5 in both conditions. These accuracy difference scores were significantly larger in the case of the acoustic verbal condition, t(72) = 2.25, p = .028, r = .26. Taken together, the analyses of online measures show a significant learning effect in both reaction times and accuracies, reflected by a decrease in reaction times and increase in accuracies through the training blocks and compared to the random reference block. This learning effect is more pronounced in the verbal acoustic than in the nonverbal visual condition.

We calculated four online learning indices for each participant in each condition in the form of residualized change scores. We built regression models with participant-level aggregated reaction times and accuracies from Blocks TRN1 and RND4 (reference blocks) as independent variables and accuracies and reaction times from blocks TRN3 and REC5 (which show the effect of learning is strongest) as dependent variables. Individual reaction time and accuracy indices were the residuals from these regression models. *RT training* was the index for residuals from a regression model that predicted median RTs in Block TRN3 from median RTs in Block TRN1. *RT difference* was calculated by averaging the residuals of regression models predicting median RTs in Block TRN3 from median RTs in Block RND4, and predicting median RTs in Block REC5 from median RTs in Block RND4. In RT indices, we inverted positive and negative signs of the values, as raw positive residual scores indicated smaller while raw negative residual scores indicated larger learning effects. Unlike *RT training*, *RT difference* is not biased by motor practice effects, as these effects are present in the random reference block, as well as the neighboring word blocks. *ACC training* and *ACC difference* were calculated in the same manner as *RT training* and *RT difference* with the exception that no change in the sign of the values was needed as raw positive residual scores indicated larger while raw negative residual scores indicated smaller learning effects.

A possible concern related to using reaction time and accuracy measures is a potential trade-off between reaction times and accuracy rates. To check potential trade-off effects between reaction time and accuracy scores, we assessed correlations between reaction time and accuracy rate pairs in each condition. In the verbal acoustic task, *RT training* and *ACC training* were positively correlated, r(74) = .28, p = .014; and *RT difference* and *ACC difference* were also positively correlated, r(73) = .49, p < .001. In the nonverbal visual task, *RT training* and *ACC training* were not significantly related, r(74) = .14, p = .244; but *RT difference* and *ACC difference* were significantly positively correlated, r(74) = .49, p < .001. These results show that there was no trade-off between online reaction time and accuracy indices, consequently, these indices can be used in subsequent analyses without such concerns.

Descriptive statistics of 2AFC overall and specific contrast scores, their differences from chance level, and their Condition effects are displayed in Table 2. All learning measures, except for the NV 2AFC word > part-word contrast, indicated significant learning effects. Performance in the verbal acoustic condition was significantly higher in all measures, except for the 2AFC part-word > non-word measure, where learning levels in the two conditions were not significantly different.

**Table 2:** Descriptive statistics of 2AFC measures, their differences from the chance level of 0.5 and their condition effects.

	verbal acoustic (VA)			nonverbal visual (NV)			Condition difference	
contrast type	mean (SD)	t or Z statistic	p	mean (SD)	t or Z statistic	p	Z statistic	p
overall	0.66 (0.13)	Z = 7.00	< .001	0.53 (0.12)	t(79) = 2.30	.024	Z = -5.57	< .001
word > part-word	0.66 (0.17)	Z = 6.31	< .001	0.50 (0.18)	Z = -0.10	.921	Z = -4.77	< .001
word > non-word	0.74 (0.19)	Z = 6.78	< .001	0.55 (0.19)	Z = 2.11	.035	Z = -5.53	< .001
part-word > non-word	0.58 (0.19)	Z = 3.54	< .001	0.55 (0.20)	Z = 2.07	.038	Z = -1.14	.253

### 3.2. Relationship between reading fluency, working memory and statistical learning

**Fig 7:**
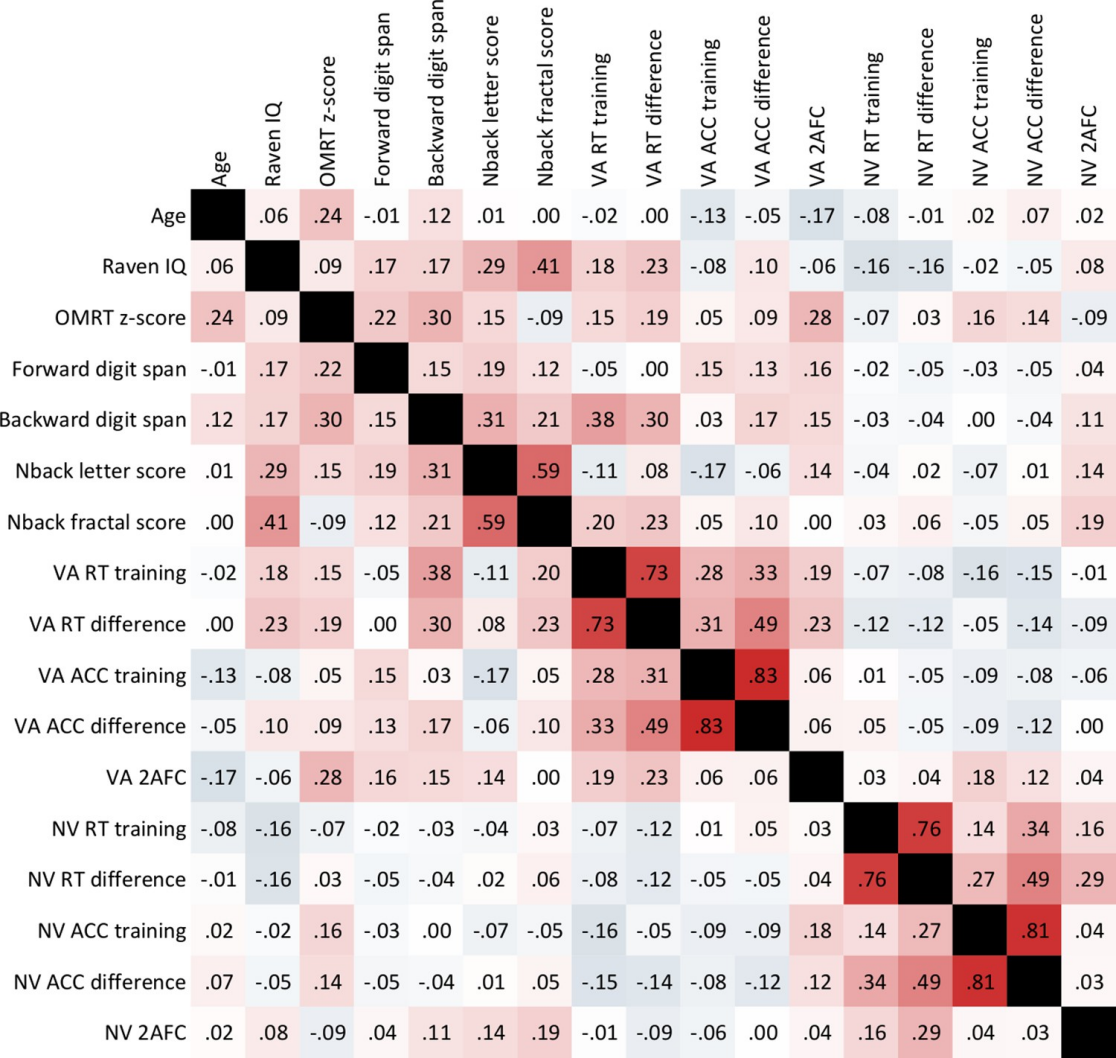
Correlations between age, IQ, reading fluency, background abilities and statistical learning measures. From the statistical learning measures, VA 2AFC was significantly related to the OMRT z-score on the level of p < .05.

We tested correlations between reading fluency, measures of verbal and visual short-term memory and working memory, and verbal acoustic and nonverbal visual statistical learning measures in the entire sample. Correlations are displayed in Fig 7.

Our primary focus of interest was the relationship between reading fluency and statistical learning measures. The testing of these questions consisted of ten correlation analyses. Although we didn’t expect the acoustic and visual statistical learning tasks to measure the same construct, we took a conservative approach and made a correction of significance levels to these ten correlations with two different methods (Holm-Bonferroni-Hochberg and Benjamini-Hochberg correction method). The correlation of reading fluency and *VA 2AFC* was marginally significant. We also conducted Bayesian correlation analyses to test the relationships between reading fluency and statistical learning measures. Correlations before and after corrections, as well as Bayes-factors are listed in Table 3.

**Table 3:** Holm-Bonferroni-Hochberg and Benjamini-Hochberg corrections and Bayes factors of the correlation analyses between reading fluency and statistical learning measures.

SL measure	r	p	rank	Holm-Bonferroni-Hochberg correction		Benjamini-Hochberg correction		Bayes factor
				corrected alpha level	corrected significance	corrected alpha level	corrected significance	
VA 2AFC	.28	.011	1	.005	non significant	.005	non significant	3.441
VA RT difference	.19	.108	2	.006	non significant	.010	non significant	0.512
NV ACC training	.16	.157	3	.006	non significant	.015	non significant	0.383
VA RT training	.15	.196	4	.007	non significant	.020	non significant	0.325
NV ACC difference	.14	.231	5	.008	non significant	.025	non significant	0.290
VA ACC difference	.09	.423	6	.010	non significant	.030	non significant	0.196
NV 2AFC	-.09	.440	7	.013	non significant	.035	non significant	0.187
NV RT training	-.07	.558	8	.017	non significant	.040	non significant	0.170
VA ACC training	.05	.694	9	.025	non significant	.045	non significant	0.155
NV RT difference	.03	.794	10	.050	non significant	.050	non significant	0.148

*Note*. Corrections of the alpha level showed that none of the relationships were significant. Bayes factors showed evidence for the relationship between the *OMRT z-score* and the *VA 2AFC* task.

## 4. Summary and discussion

The present study provides new results on statistical learning abilities in individuals with different levels of reading proficiency: we investigated segmentation abilities as a function of reading fluency in both the acoustic verbal and visual nonverbal modalities. Together with post-training 2AFC measures, we also employed an online target detection task [70] to obtain measures of statistical learning which are less confounded by metacognitive skills and are able to track learning in the course of the training. All participants were tested in both the visual and verbal modalities. This design allowed us 1) to investigate statistical learning as a function of reading abilities in different domains and modalities, 2) to compare the efficiency of online and offline statistical learning measures, and to investigate the relationship between reading fluency and online and offline statistical learning measures.

Throughout the verbal acoustic and nonverbal visual conditions, reaction times and accuracies showed significant learning effects in the target detection task, reflected in reaction time and accuracy changes through the online blocks. This learning effect was larger in the verbal acoustic condition. The learning effect measured by the 2AFC task was significant as well in both conditions, with a larger effect in the verbal acoustic condition. From the statistical learning measures, only the *verbal acoustic 2AFC* index showed a relationship with reading fluency scores: better reading skills were associated with higher scores in the verbal acoustic offline task. However, correcting for multiple comparisons yielded a nonsignificant association even for this measure.

According to our first hypothesis, we expected that statistical learning would be significant and equally efficient in the verbal acoustic and the nonverbal visual conditions. We found significant learning effects in both conditions in the online tasks, and participants performed above chance level in the offline 2AFC task in both conditions. However, we found more pronounced learning effects in the verbal acoustic condition in both online and offline tasks. In line with our second hypothesis, we found significant learning effects in both online and offline measures. Our third hypothesis on the association between statistical segmentation and reading fluency in both verbal acoustic and nonverbal visual conditions was not supported, as only one verbal acoustic index was significantly related to reading fluency, which was no longer significant after correcting for multiple correlations. Based on our fourth hypothesis, we expected both online and offline measures to be associated with reading fluency in both modalities, with a stronger association with online ones. Again, this hypothesis was not supported, as only the offline 2AFC measure of the verbal acoustic task showed a significant positive relationship with reading fluency, and this association was no longer significant after controlling for multiple comparisons.

While there are several studies supporting the association between statistical learning in nonverbal visual and visuomotor tasks and reading skills in both typical development and in dyslexia [26, 47, 53, 84–88], similarly to other studies [4, 22, 88, 89], we did not find a significant relationship between visual statistical learning and reading deficits. Although after correcting for multiple comparisons, our finding of the association of reading fluency with verbal acoustic statistical learning capacity was no longer significant, together with previous findings of a positive association [63], they emphasize the need for further investigating the effect of acoustic statistical learning in reading skills. The importance of acoustic statistical learning might be especially pronounced in the case of shallow orthographies like Hungarian. As in such systems, grapheme-phoneme correspondences are consistent, and there are few irregularities, readers in shallow orthography systems may rely primarily on the phonological route in reading compared to more opaque orthography systems, and knowledge about phoneme patterns extracted through statistical segmentation might contribute more strongly to reading abilities [66–68]. However, testing this relationship at earlier ages when reading skills are still developing may yield better insights into the relationship of statistical learning and reading skills (both in the visual and the acoustic modality).

Differences in associations of reading skills with various measures in different studies could result from differences in task design and sensitivity indices. We aimed to employ a diverse set of measures with both online and offline indices, and we also aimed to improve the sensitivity of previous 2AFC tasks. Online tasks measure learning more directly without relying on metacognitive awareness of the learned structure and are not confounded by other abilities, and, as a result, they might show evidence of learning even in cases where offline measures fail to do so (as reflected in the difference in the number of learners in the 2AFC versus all online measures in the nonverbal visual task). At the same time, the design of our online task tested differences only between reactions to a single target syllable in a word versus reactions to the same target syllable in random sequences. In contrast, the 2AFC task tested discrimination in more diverse settings, requiring choosing between words versus part-words, words versus non-words (random sequences) and part-words versus non-words, covering a larger and more variable range of participants’ potential knowledge about words and transitional probabilities. This methodological difference may result in online and offline tasks measuring different aspects of statistical segmentation. For example, online tasks may target item-item relationships, while the 2AFC tasks also test memory of larger patterns; or online and offline tasks may examine implicit and more explicit memory or metacognitive knowledge of the structure, respectively.

Besides task design (online versus offline), the characteristics of the input may also pose different constraints on learning and yield more efficient learning on one or the other learning measure. Sequential learning of nonverbal visual patterns of symbols may be more difficult than sequential learning of verbal acoustic patterns. Developing metacognitive knowledge may be easier for verbal than for nonverbal strings; this may explain lower performance on the nonverbal visual, but not the verbal acoustic tasks.

Our study is not without limitations. Participants’ reading capacities were determined based on reading fluency as measured by a reading-aloud task, but we did not test e.g., reading comprehension or silent reading. Relying on a more complex measure would have definitely given us a better picture of reading abilities, allowing a more accurate measurement of the relationship with statistical learning. The choice and design of the segmentation task also raise potential methodological issues. One could argue that segmenting four three-syllable words might have been too easy for participants in this age group, even for participants with potential statistical learning deficits. This is countered by a continuous improvement in online learning during the three training blocks in both modalities, and far from ceiling performance in the offline measures. Notwithstanding, further studies with both offline and online statistical learning tasks better suited for testing individual variation by including items differing in complexity could possibly yield a different pattern. Our study was an attempt to address some of these methodological issues, but future studies with various designs and stimuli using better measures could also improve sensitivity of both online and offline measures [69, 71, 90].

Statistical learning is a multi-faceted capacity with not only modality and domain specific, but also stimulus and structure dependent constraints. Recently, Bogaerts and colleagues [1] emphasized the need for specific hypotheses on the nature of the statistical learning deficit and the language symptoms observed in language impairments, including reading difficulties. In line with this suggestion, we aimed to test segmentation abilities which are relevant in learning to read in both the acoustic and visual modality. We believe that our work is a relevant contribution to research on the relationship between statistical learning and reading, and, by employing both online and offline measures, to the larger field of statistical learning as well. Together with results and controversies from previous studies, these findings call for further and more targeted and specific hypotheses concerning this relationship. These should relate statistical learning of patterns directly involved in reading (e.g. letter cooccurrences and grapheme-phoneme mappings) to more complex reading measures that focus on various aspects of reading beyond fluency (see [1]), to see whether visual statistical learning of e.g. letter combinations is related to reading.

## 5. Conclusion

In the present study, we investigated the relationship between reading ability and statistical learning. We observed efficient learning in both the acoustic and visual statistical segmentation tasks in both online and offline measures, showing that the online target detection task is a promising tool in investigating statistical learning. We haven’t found evidence for a significant relationship between statistical learning and reading fluency in adolescents in either modality. The controversial set of findings from different studies together with our results suggest that the relationship between reading and statistical learning deficit may be dependent on the domain, modality and nature of the statistical learning task, on the reading task, on the age of participants, and on the specific language. For this reason, further studies are needed to clarify how statistical learning contributes to written and oral language skills, and whether its deficits are causal in the reading difficulties, or accompany reading difficulties without contributing to them.
